# 

**Published:** 1874-01-15

**Authors:** 


No. II. CHICAGO, JANUARY 15, 1874. Vol. XV.

SURG. GEN'L'S OFFICE. LIBRARY.

### THE Medical Examiner.

A

### Semi-Monthly Journal of Medical Sciences.

*TERMS.— Yearly Subscriptions, Three Dollars, in advance. Single Number Fifteen Cents. All Communications should be addressed to Publishers* MEDICAL EXAMINER, 65 *Randolph st., cor. State, Chicago.*

